# Environmental Tobacco Smoke Exposure and Smoke-Free Rules in Homes among Socially-Disadvantaged Populations in Poland

**DOI:** 10.3390/ijerph14040447

**Published:** 2017-04-21

**Authors:** Katarzyna Milcarz, Leokadia Bak-Romaniszyn, Dorota Kaleta

**Affiliations:** 1 Department of Tobacco Control, Preventive Medicine Department, Medical University of Lodz, 90-752 Lodz, Poland; dkaleta@op.pl; 2 Department of Nutrition in Digestive Tract Diseases, Medical University of Lodz, 93-338 Lodz, Poland; leokadia.bak-romaniszyn@umed.lodz.pl

**Keywords:** tobacco control, environmental tobacco smoke, smoke-free home, low socioeconomic status population

## Abstract

This study aims to examine the prevalence of exposure to environmental tobacco smoke (ETS) in homes among socially-disadvantaged populations in Poland, along with the prevalence and correlates of voluntary implementation of smoke-free home rules. Data concerning 1617 respondents from a cross-sectional study completed in the Piotrkowski District were used, which was part of the “Reducing Social Inequalities in Health” program. Overall, 19.4% of the respondents declared exposure to ETS at home. In the non-smokers group, 15.5%, including 6.6% males and 18.3% females, were exposed to ETS in their place of residence (*p* < 0.0001). Complete smoke-free rules were adopted by 22.1% of the study participants. Two factors, smoker status and lack of ETS-associated health risk awareness, were found to be significantly associated with no adoption of total smoking bans at home. Socially-disadvantaged non-smokers, especially females from rural areas in Poland, still constitute a large population exposed to ETS in their homes—a challenge from the perspective of public health. Focused efforts are required to address social norms around exposing others to ETS.

## 1. Introduction

Environmental tobacco smoke (ETS) contains more than 7000 harmful chemicals, a combination of which makes it highly toxic, resulting in cancer in humans [1,2]. Exposure to ETS causes death, disease, and disability [3,4,5]. Studies have confirmed that environmental tobacco smoke exposure increases the risk of lung cancer in adults, breast cancer in (primarily pre-menopausal) women, heart disease and heart attacks, and asthma induction and exacerbation [6,7,8,9]. Some of the most vulnerable groups affected by ETS include infants and children [10,11,12], but also socially-disadvantaged populations in relation to which it has been shown that tobacco is a major contributor to the differences in mortality between the least and most advantaged [13].

It is estimated that ETS exposure contributes to about 1% of the total global disease burden, and represents approximately 10%–15% of the disease burden caused by active smoking [10]. In addition to an already large and growing health burden, ETS exposure also has a significant economic impact on individuals and countries, both in relation to the costs of direct health care, as well as the indirect costs resulting from reduced productivity [14,15]. It is also estimated that 10% of the total tobacco-related economic costs are attributable to ETS exposure [16]. The disadvantaged groups are bearing a disproportionate share of the harm caused by tobacco, including higher rates of death and disease from tobacco-related illnesses [17,18].

According to the World Health Organization (WHO), adoption of completely smoke-free environments is the effective way to protect human health from the harmful effects of ETS, as there is no safe level of exposure [8,10].

Studies of the effects of smoke-free policies have demonstrated that their consistent implementation leads to a decrease in exposure to second-hand tobacco smoke by 80%–90% in high-exposure settings, and that such policies can lead to an overall decline in exposure of up to 40% [19,20]. People working in smoke-free environments are significantly less exposed to ETS than other workers [21]. Smoke-free environments not only protect non-smokers, but they also positively affect tobacco use in continuing smokers by helping them to reduce the number of cigarettes smoked daily and encouraging them to quit [10].

In addition to implementing smoke-free work and public environments, voluntary smoke-free home policies may have some potential to reduce the tobacco-related harm. Non-smoking adults residing in comprehensive smoke-free communities are up to 10 times less likely to be exposed to second-hand tobacco smoke than those living in areas where no smoke-free legislation is in place [22,23]. It has been shown that smoke-free homes also decrease adult and youth ETS exposure and smoking, as home smoking bans reduce progression to smoking experimentation among young people who live with non-smokers [24]. Teenagers residing in homes where smoking is allowed are nearly twice as likely to start smoking, even if the adults are non-smokers themselves, as compared to homes where smoking is prohibited [24]. Moreover smoke-free homes have been associated with reduced smoking behaviors among adult smokers [25,26,27].

A review performed by Mills et al. showed that smoke-free homes may disrupt established smoking patterns such that individuals may smoke less or delay their first cigarette of the day because of the inconvenience of having to go outside to smoke [26]. This may encourage smoking reduction or cessation attempts. Among those who have quit, a smoke-free home may prevent relapse because of the absence of environmental cues to initiate smoking (e.g., other smokers in the household) [26]. As demonstrated in study by Vijayaraghavan et al. smoke-free homes may be a more important intervention than price to encourage successful quitting in low-income smoker [28].

On the other hand, lower-income adults have been shown to be less likely than higher-income adults to adopt smoke-free homes, and more opposed to ETS, reflecting differential smoking norms in the respective communities [27,29,30]. Previous studies revealed that apart from income several factors are associated with individuals’ decisions to adopt smoke-free homes, including educational attainment, age, gender, smoking status, living with a partner, cohabitation with a smoker or children, awareness on ETS-related health risks, workplace smoking policy in indoor areas, housing-type, etc. [29,31,32,33,34]. As has been shown in various studies, the prevalence of smoke-free home rules observed nationally and across countries may be attributable to multiple factors, including the spread of national and local comprehensive smoke-free laws prohibiting smoking in public areas and workplaces, declining smoking prevalence among adults and teenagers, as well as to population-level interventions and activities leading to children’s and disadvantaged populations’ ETS exposure at home [1]. The smoke-free homes adoption level differs from country to country, with the US taking the lead with over 80% of the population declaring having adopted complete home smoking bans (90% among non-smokers and 50% among smokers) [35,36,37,38]. In European countries, on the other hand, complete home smoking bans were reported by 59.5% of the French, 63.5% of the Irish, 61.3% of the Italian, 74.4% of the Czech, and 87% of the Swedish females in 2010 [39]. Once all of those European data are compiled, the prevalence of home smoking bans varies between smokers and non-smokers [39], with the reported adoptions among 75% of non-smokers and 50% of smokers. Sweden had the largest proportion of participants who reported having implemented smoke-free homes, including both smokers and non-smokers [39]. Among non-smokers, Italy demonstrated the lowest total smoking ban adoption level (66%), and among smokers, France stood out with the lowest proportion of smoking restrictions implemented at home (31%). In Georgia in a 2014 national survey, only 14.3% of the respondents reported complete smoke-free home policies. China also showed a low total smoking ban at home adoption level, with 9.3% of the households admitting to having implemented a full smoking ban, 12.1% a partial ban, and 78.6% of the households having no restrictions whatsoever [40].

Even though Poland has implemented legal limitations on smoking or complete smoking bans in public areas and selected workplaces, not as much effort has been implemented to encourage the adoption of smoke-free rules in private settings. Further, there is insufficient research in the area of ETS exposure at home and very little information on the adoption of smoke-free home rules in Poland. Specifically, the relevant data are currently not available for persons originating from socially-disadvantaged environments.

It is a known fact that the growing disparity in smoking rates contributes to increased social stigma and marginalization as smoking becomes less accepted in the general community, and is also contributing to financial inequalities [41]. At the same time, one must not forget that tobacco contributes to poverty through the cost of tobacco-related illness, including ETS-related harm, loss of a family breadwinner, and the impact on family finances and stress. [17,42]. With all of these concerns in mind, this study aimed to examine the prevalence of exposure to ETS in homes among socially-disadvantaged subpopulations in Poland, along with the prevalence and the factors associated with voluntary implementation of smoke-free rules at home.

## 2. Materials and Methods

### 2.1. Sample and Study Design

The present study is based on the data collected during a cross-sectional study completed in the Piotrkowski District, which was part of the PL-13 Program, “Reducing Social Inequalities in Health”, supported by a grant from Norway through Norway Grants and co-financed by the Polish state. The project was popularized under the official name of “Your Heart is Your Life” [43]. The enquiry involved a total sample of adults aged 18–59, socially-disadvantaged residents of the Piotrkowski District, registered with the local government welfare assistance institutions and entitled to receive social aid. Social aid in Poland is administered by the local and national governments [44]. The current form of social aid has been in place since 1 May 2004 [44]. The right to benefits under the system is given to individuals and families who are unable to cope with difficult life situations using their own empowerment, resources and abilities [44]. Under the law assistance to the person in need is mandatory and no family should live below a certain income level. The basic criterion for receiving the social monetary benefits is the level of monthly income per capita in the household. Per the relevant Social Care Act and for the purposes of the study, socioeconomically-disadvantaged (SD) persons were defined as those receiving a minimum income not greater than $158 USD (634 PLN—Polish currency) per month in relation to single persons, and not greater than $128 USD (514 PLN—Polish currency) per month in relation to family members [44]. The research covered all those who met the inclusion criteria and agreed in writing to participate in the study. Written informed consent was obtained from all study participants, while the Bioethics Committee of the Medical University in Lodz positively reviewed the project (Project Identification Code: RNN/243/15/KE). Detailed characteristics of the Piotrkowski District, the program assumptions, and the methodology have all been published elsewhere [43].

According to the state as of the year 2013, there were 91,618 residents, including 45,223 men and 46,395 women, living on the premises of the Piotrkowski District with more than 90% of the residents representing rural areas. In 2013, in Piotrkowski District, approximately 9% of its residents required the support of social assistance institutions due to the lack of resources to live on, and poverty concerned 11,867 people in 4336 families [43]. In the group of 11,867 social care beneficiaries there were 3636 of inhabitants aged 18–59. The entire population of beneficiaries of government welfare assistance aged 18–59 from Piotrkowski district was surveyed. Finally, data from close to 50% of representatives of this subpopulation were obtained. In total, 1817 face-to-face interviews were completed between October 2015 and February 2016 among the target population, with an overall response rate of 49.97%. The relevant dataset can be found in the supplementary materials (Table S1: SM1). The age and the gender distribution of respondents has been checked and no statistically significant difference of the age and gender structure in the group of respondents compared to non-respondents group has been found.

### 2.2. Measurements

The questionnaire details are available for review in the previous research paper by Milcarz et al. [43]. The study gathered information on the respondents’ socio-demographic background, including gender (male or female), age (years), educational attainment (primary, vocational, secondary, and higher education), subjective assessment of living conditions (fair, rather fair, neither fair nor poor, rather poor, poor, do not know), marital status (single, married, divorced, or widowed), employment status (currently employed with a permanent job, temporarily employed, retired, disability pensioners, students, currently without a permanent or part time job, or unemployed), subjective assessment of monthly income (sufficient to cover all living needs and able to save a certain amount, sufficient to cover all living needs, sufficient to cover the basic needs only, not sufficient to cover even the basic needs, response declined, don’t know), subjective assessment of health condition (fair, rather fair, neither fair nor poor, rather poor, poor), and the respondents’ declared health problems (none, between 1 and 3, between 4 and 6, more than 7), and cohabitation with partner and/or family (no-living alone, yes). Data on tobacco smoking status, exposure to ETS, and implementation of smoke-free rules at home were also collected, while the awareness of ETS-related health risks was investigated.

The current smokers category comprised two subgroups: the daily smokers (smoking one or more cigarettes per day during the past 30 days at a minimum) and the occasional smokers [43]. Respondents were asked if they had been exposed to ETS 30 days prior the study, with the available answers including: “yes, at home only”; “yes, at work only”; “yes; both at work and at home”; “yes, in other places only”; “no, not at all”. Respondents exposed to ETS at home were considered those answering “yes, at home only” and “yes; both at work and at home”. Smoking restrictions in the home were assessed based on the question: “Which of the following best describes smoking rules in your home?”. One of the available responses was: “Not allowed at all in any of the indoor premises” which automatically classified the respondent as “having a smoke-free home”. However, respondents who reported that smoking was allowed in some or all areas of the home or answered “do not know”, were classified as lacking a smoke-free home. The total ETS exposure (number of hours of daily exposure to tobacco smoke at any place, including home, work, and public places) was classified as none, <1 h, 1–5 h, 5–8 h, and more than 8 h per day. Apart from the above, the study further assessed the respondents’ awareness of ETS-related health risks. The awareness of the ETS-related health risks was assessed based on the question “Do you think that ETS causes serious diseases?”, with the possible answers “yes, no, do not know”. Respondents were, thus, categorized as “aware of the negative health consequences” (positive response to the above question), and “unaware of the negative health consequences” (the negative and the “do not know” responses to the above question).

### 2.3. Statistical Analyses

An extended Mantel-Haenszel chi-squared test was used to analyze associations between categories of all examined variables and implementation or no of smoke-free homes. The univariable and multiple logistic regression analysis was implemented to calculate the odds ratios (ORs) and the 95% confidence interval (CI) of each variable in relation to the lack of implementation of smoke-free home rules. Initially, crude coefficients—ORs of the impact of odds variables on the lack of implementation of smoke-free home rules were calculated. The variables used to adjust the models were: gender, smoking status, subjective assessment of living conditions, education, enployment status, awareness on environmental tobacco smoke health consequences, subjective assessment of monthly income, subjective health state, and cohabitation with a partner and/or family. This was followed by a multifactorial analysis considering the simultaneous effect of all of the variables on the risk of the lack of total smoking bans at home. The multiple logistic regression analyses included all of the variables significantly associated with a lack of implementation of smoke-free home rules in any of the univariate models (*p* ≤ 0.05). All *p* values were two-sided and *p* < 0.05 was used to indicate the statistical significance. The STATISTICA Windows XP version 10.0 program (StatSoft Poland Inc., Tulusa, OK, USA) software package was used to complete the statistical analysis.

## 3. Results

### 3.1. Characteristics of the Study Population, Prevalence of ETS Exposure, and Adoption of Smoke-Free Home Rules

Of the 3636 beneficiaries of social assistance sampled in the study, 1817 persons, including 1224 females and 593 males, participated in the research and finalized their interviews. However, for the purposes of the current analysis, data from 1617 (1091 females and 526 males) fully completed questionnaires were used, while records containing missing information were disregarded. Table 1 shows the characteristics of the study group, the prevalence of ETS exposure at home, and the adoption of smoke-free homes rules. Overall, 19.4% (n = 313) of the respondents declared exposure to ETS at home, including 17.1% (n = 90) of males and 20.4% (n = 223) of females (*p* > 0.05). In the non-smoker group, 15.5% (n = 157), including 6.6% (n = 16) of males and 18.3% (n = 141) of females, were exposed to ETS in their place of residence (*p* < 0.0001). In the smoker group, 25.8% (n = 156), including 25.1% of males (n = 74) and 25.5% (n = 82) of females, were exposed to ETS in the past month (*p* > 0.05). The ETS exposure rates in the place of residence among smokers and non-smokers, and in relation to the implementation of total smoke-free rules at home, or lack of them, have been presented in Table 1.

The study further investigated the tobacco smoking restrictions at home (within the residential premises of the respondents’ homes), with complete smoke-free rules adopted by 22.1% (n = 358) of the study participants. The prevalence of total smoking bans at home was declared by 20.3% of male (n = 107) and 23.0% (n = 251) of female respondents (*p* > 0.05). In the non-smokers group, 25.5% (n = 258), including 23.9% (n = 58) of males and 26.0% (n = 200) of females (*p* > 0.05) implemented total smoking bans in their place of residence. Among smokers, 16.5% (n = 100), including 17.3% (n = 49) of males and 15.9% (n = 51) of females (*p* = 0.1), implemented 100% smoke-free rules at home. The prevalence of smoking restriction implementation at home by smoking status and in relation to selected characteristics have been shown in Table 1.

### 3.2. Associations of No Implementation of Total Smoking Bans at Home

The results from the logistic regression analyses have been presented in Table 2. Compared to non-smokers, smokers were significantly more likely to live in houses where complete smoking bans had not been implemented (OR = 1.71; 95% CI: 1.32–2.21; *p* < 0.001). The odds ratios (OR) and the 95% confidence intervals (CI) for lack of total smoking ban implementation at home showed that the respondents who were unaware of ETS-associated health risks had much higher odds of lacking smoke-free homes compared to those aware of such threats (OR = 1.28; 95% CI: 1.00–1.65; *p* < 0.05). Other variables in the analysis were not significantly associated with the lack of smoke-free home rules.

## 4. Discussion

A cross-country comparison revealed that the prevalence of ETS exposure among adults at home varies in different countries, ranging from 73.1% in Vietnam, 67.3% in China, 62.5% in Egypt, 54.9% in Bangladesh, to 34.7% in Russia, 33.2% in Thailand, 27.9% in Brazil, 23.5% in Ukraine, and 17.3% in Mexico [45,46]. Several studies have also highlighted the higher likelihoods of ETS exposure at home for socioeconomically-disadvantaged people [46,47,48,49]. The present study has documented that, even though the smoking prevalence among social aid recipients tends to be higher [43], the prevalence of ETS exposure for the population of the Piotrkowski District was found to be much lower than in the general population, where 15.5% of the non-smokers (6.6% of males and 18.3% of females, respectively) were exposed to ETS in the month preceding the study. This shows that ETS exposure is currently smaller by almost half in relation to the general population Global Adult Tobacco Survey (GATS) results completed six years earlier, where ETS exposure at home at least once a month was reported by 28% of the respondents, including 24.9% of non-smoking males and 30.4% of non-smoking females. On the one hand, this result may be due to the specific living conditions in the countryside where most residents occupy single-family houses with private yards, making it much easier for smokers to leave the covered premises and have a cigarette/no smoking bans than in the case in cities where most people live in multi-apartment buildings, and spaces to have cigarettes outside of the home are more difficult to find. In addition, country dwellers, in general, spend more time outdoors/outside of the covered premises to carry out work associated with running a household or farm, or performing a job which is mostly related to agriculture, horticulture, animal husbandry, etc., which do not set any physical limits to smoking. However, a lower level of ETS exposure, as compared to the national indicators, may also reflect changes (taking place between 2009–2016) coinciding with the implementation of the smoke-free legislation, social norms shifting in favor of the ban, with the subsequent decrease in ETS exposure. Unfortunately, no ETS exposure data for 2009–2010 are available for socioeconomically-disadvantaged people living in rural areas to be able to assess if a positive change has taken place, or if the observed differences are due to varying frequencies of ETS exposure at home for the SD people compared to the general population or persons coming from high socioeconomic backgrounds. Further study is required to identify the influencing factors.

Nevertheless, compared to high-income countries, such as the United States, the prevalence of the residential ETS exposure in the rural areas of Poland was approximately three times as high as that found in the US in 2009 (6%) and the self-reported household exposure (4%) in the 2010 NHANES population [35,50]. This probably reflects the differences in the comprehensiveness of tobacco control measures implemented in the respective countries, as well as the social approval for smoking.

One of the most important findings of this study was that ETS exposure was three times more prevalent at home for non-smoking women (18.3%) than for men (6.6%) from Piotrkowski District. This is also consistent with other studies in the area [47,51].

The declared level of total smoking bans within closed premises at home was relatively low, with only 22.1% of the respondents residing in the Piotrkowski District admitting to having implemented smoke-free homes. Earlier data based on the GATS 2009–2010 study indicated an adoption level of 37.1% [52]. Similar to ETS exposure, there is no previous comparative data comprising the SD population from rural areas, nor is there any current research available covering the general population which would demonstrate the total smoking ban adoption level for private homes.

Multivariable analyses have indicated two significant correlates of the smoking at home allowance, i.e., smoker status and lack of ETS-related health risk awareness. The higher correlates of allowing smoking at the homes of smokers (vs. non-smokers) seems obvious enough and has been corroborated by other research in the area [52,53,54]. For instance, a study by Kaleta et al. showed an approximately two times lower likelihood of adopting total smoking bans at home among current smokers (both male and female) compared to non-smokers in Poland [52]. Heck et al. correspondingly found that adopting a complete smoking ban at home was correlated with the smoking behaviors and demographic characteristics of study participants [39]. The present findings are further supported by the results of a study by Gonzales et al. on the prevalence and predictors of home smoking bans among US women with young children, which revealed that current smoking and cohabitation with other smokers were associated with an increased likelihood of not having implemented complete smoking bans [55]. Previous studies have also shown that Chinese parents tend to be unaware of the health risk of ETS exposure, while the main reasons for not adopting smoking bans at home included the social acceptability of smoking and the predominant influence of male family members. Awareness of the harmful effects of smoking and ETS exposure has been associated with the adoption of home smoking bans in China [40,56,57]. Similar to a previous study conducted in Poland among economically-active respondents by Kaleta et al., low awareness of the health consequences of ETS turned out to be the significant predictor of the total smoking ban adoption level among the residents of the Piotrkowski District [52]. Those unaware of the adverse health effects of ETS were approximately two times less likely to adopt smoke-free homes versus those perceiving ETS as harmful. A systematic review by Passey et al. listed numerous barriers to adopt smoke-free homes, including poor awareness and knowledge of the risks from ETS among the most important [58].

It appears that smoke-free home rules have yet to become the focus of public health experts in Poland with the study results representing valid proof that continued health awareness education and campaigns in favor of introducing complete smoking bans in the home need to be offered to underprivileged populations not only in the Piotrkowski District, but across the entire country.

Given the large numbers of nonsmokers who are at risk of exposure in private settings, there is a need for effective tobacco control strategies to protect those most vulnerable [58]. The study results underscore the need for interventions to encourage the adoption of smoke-free homes among low-income populations, with the findings offering insight for policy-makers to develop measures for the effective implementation of interventions aimed at increasing smoke-free home rules.

These measures may include public health interventions to increase the regulation of secondhand smoke in public housing, or educational interventions to improve restriction adoption among low-income populations [58]. Opportunities for delivering targeted messages to households and communities include mass media campaigns, printed resources and guidelines, as well as individual interactions between smoking households and health professionals and other stakeholders offering support to smoking households). Preliminary evidence shows that media campaigns specifically targeting secondhand smoke have been effective at reducing smoking in the home, while initiatives focusing solely on smoking cessation have not been as successful [58]. Professionals should give specific and evidence-based risk messages about ETS exposure to households, particularly focusing on hazards affecting pregnant women, babies, and older children. These messages need to make it very clear that there is no safe level of ETS exposure, explain what a smoke-free home is, how to make and maintain one, and what the potential benefits of having a completely smoke-free home are. This includes transparency concerning the lack of efficacy of strategies such as burning candles and opening windows. It should be further stressed that all adult members in a household, rather than specific individuals, for example mothers, have the responsibility for establishing and maintaining a smoke-free home. As households cannot be required to go smoke-free by legislation, clinical and educational initiatives are the only viable direct approaches for reducing SHS exposure in this setting [59]. In interacting with smoking households, health professionals should always consider SHS exposure and SFH in addition to smoking cessation. For example, if a smoker responds negatively to cessation advice, the professional should strive to inquire into areas such as SHS exposure and SFHs. This exploration should include current home smoking rules and whether these are flexible and why. Within these discussions, professionals need to respect and recognize the complexity and challenging circumstances faced by some households, and should aim to prevent further stigmatization of smoking households whose members are often doing their best to respond to adversity. The barriers, motivators, and enablers of smoke-free home are likely to be unique to each household, in part relating to social and cultural norms relevant to that household. As such, health professionals should build on the positive changes people have already made in their homes, including, for example, not smoking in the home following the birth of a baby. They might further seek to reassure households that others have created and maintained SFHs while successfully protecting important relationships within their family and social networks. Education and training of health professionals should include skills development in the area of advising on SFH-related practical strategies, such as overcoming weather-related barriers, to support households in having and maintaining a SFH.

## 5. Study Limitations and Strength

The current study has several strengths. First of all, for the first time a study on the prevalence and factors associated with ETS exposure and smoke-free homes has been conducted among a socially-disadvantaged population in Poland. Secondly, from all those invited to participate in the study, approximately one half agreed to take part which makes the rate comparable to the one obtained in other similar surveys in Poland (60% in the GATS Poland study and 45% in the Multi-Centre National Population Health Examination Survey II study) [60,61]. Finally, the interviewer-administered questionnaires completed during face-to-face interviews resulted in higher sensitivity and specificity values, compared to self-administered questionnaires, and helped to reduce the non-response rate [43]. However, specific study limitations, already broadly discussed in a previous research paper, need to be highlighted [43]. Firstly, ETS exposure assessment was based on self-reports, which may have led to misreporting. Second, smoke-free home rules were also self-reported, which could also introduce some bias. Thirdly, a limitation with the lack of certain questions existed in the questionnaire which could have been relevant for in-depth analysis, such as questions on cohabitation with a smoker, children, and other questions of socioeconomic and demographic characteristics. Fourthly, reports on the smoke-free home rules were collected from a single member of the household and no data were available to examine the validity of individual-level reports of home smoking restrictions at the household level [62]. Another limitation concerns the fact that the data do not include information regarding the relative enforcement or the timing of the ban implementation, both pertinent to anticipating health outcomes, which may lead to a generalizability of the findings. As has been shown in a study by Mumford et al. discrepancies between individual educational attainment levels, expected to be closely related to individual smoking status, may help explain the discrepancies in the perception (or enforcement) of home smoking bans. Multi-member households with smokers were substantially less likely to consistently report strict home bans. One in four households (and three in five households with children) has a nearly 60% higher probability of discrepant reports and, thus, some level of secondhand smoke exposure. These findings suggest that the problem of discrepant reports from multi-member households, especially where there is a current smoker or an ex-smoker and children, is large enough to merit closer examination of the targeted messages. It has also been suggested that public policies may influence the consistency of individual views by providing information about the nature and effects of home bans. It is possible that comprehensive media policies may clarify what constitutes a home ban [62]. Potentially, also, is that the respondents may have misclassified the presence of smoke-free rules in their homes. Implementation of objective measures of tobacco smoke exposure might be useful in further studies [63,64,65,66]. The cross-sectional design is another limitation of the study as it tends to make observations at a single point in time, which prevents the observation of changes in ETS exposure or smoke-free homes enforcement over longer periods of time [43].

## 6. Conclusions

Socially-disadvantaged non-smokers, especially females living in rural Poland, still constitute a large population exposed to ETS in their homes, which is challenging from a public health perspective. Moreover, low levels of the adoption of smoke-free rules is observed. Intensive interventions targeting especially male smokers, are required for achieving further progress in the reduction of ETS exposure. These findings should urge policy-makers and relevant stakeholders at the local level to take into account ETS exposure at home and smoke-free home issues while planning and implementing tobacco control measures in rural communities. Efforts to address social norms around exposing others to ETS and increasing awareness of the health consequences of ETS combined with advice on the benefits of smoke-free living, should become the top priority. However, prevention efforts for smoking uptake among socially-disadvantaged rural populations in Poland, and strengthening cessation services to help smokers quit, should not be neglected. Improved continued monitoring of ETS exposure and smoke-free homes is required. Furthermore, continuing surveillance among general and vulnerable populations is necessary to evaluate progress towards the reduction of ETS exposure in various settings, including workplaces, public places, and private environments.

## Figures and Tables

**Table 1:** Characteristic of the study participants, environmental tobacco smoke exposure, and smoke-free rules in homes among socially-disadvantaged residents of Piotrkowski District.

Characteristic	Total Sample		*p*-Value *	Smokers		*p*-Value *	Non-Smokers		*p*-Value *
	Total Ban	No Total Ban		Total Ban	No Total Ban		Total Ban	No Total Ban	
	n (%)	n (%)		n (%)	n (%)		n (%)	n (%)	
Overall	358 (22.1%)	1259 (77.9%)		100 (16.6%)	504 (83.4%)		258 (25.5%)	755 (74.5%)	
Gender	358 (22.1%)	1259 (77.9%)		100 (16.6%)	504 (83.4%)		100 (16.6%)	504 (83.4%)	
Men	107 (20.3%)	419 (79.7%)	>0.05	49 (17.3%)	234 (82.7%)	*p* > 0.05	58 (23.9%)	185 (76.1%)	>0.05
Women	251 (23.0%)	840 (77.0%)		51 (15.9%)	270 (84.1%)		200 (26.0%)	570 (74.0%)	
Age (years)									
<30	48 (26.5)	133 (73.5)	>0.05	13 (20.6)	50 (79.4)		35 (29.7)	83 (70.3)	>0.05
30–39	143 (20.6)	550 (79.4)		37 (16.4)	188 (83.6)		106 (22.7)	362 (77.4)	
40–49	120 (22.1)	422 (77.9)		35 (15.6)	189 (84.4)		85 (26.7)	233 (73.3)	
50–59	47 (23.4)	154 (76.6)		15 (16.3)	77 (83.7)		32 (29.4)	77 (70.6)	
Education									
Primary	86 (19.1)	362 (80.8)	>0.05	35 (15.4)	192 (84.6)	>0.05	51 (23.0)	171 (77.0)	>0.05
Vocational	119 (22.0)	421 (78.0)		41 (18.2)	184 (81.8)		78 (24.8)	237 (75.2)	
Secondary	128 (23.5)	417 (76.5)		24 (16.4)	122 (83.6)		104 (26.1)	295 (73.9)	
High	25 (30.1)	58 (69.9)		0 (0.0)	6 (100.0)		25 (32.5)	52 (67.5)	
Subjective assessment of living conditions									
Fair	49 (28.3)	124 (71.7)	<0.01	17 (28.8)	42 (71.2)	*p* > 0.05	32 (28.1)	82 (71.9)	<0.05
Rather fair	134 (22.9)	452 (77.1)		29 (14.9)	166 (85.1)		105 (26.8)	286 (73.2)	
Neither fair nor poor	154 (21.4)	567 (78.6)		48 (17.7)	223 (82.3)		106 (23.6)	344 (76.4)	
Rather poor	11 (14.5)	65 (85.5)		6 (13.0)	40 (87.0)		5 (16.7)	25 (83.3)	
Poor	0 (0.0)	29 (100.0)		0 (0.0)	19 (100.0)		0 (0.0)	10 (100.0)	
Difficult to say	10 (31.3)	22 (68.7)		0 (0.0)	14 (100.0)		10 (55.6)	8 (44.4)	
Cohabitation with partner and/or family									
No (living alone)	55 (21.5)	201 (78.5)	>0.05	19 (16.7)	95 (83.3)	*p* > 0.05	36 (25.4)	106 (74.6)	>0.05
Yes	303 (20.1)	1058 (79.9)		81 (11.7)	409 (88.3)		222 (24.6)	172 (75.4)	
Employment status									
Permanent job	107 (23.5)	348 (76.5)	>0.05	32 (21.9)	114 (78.1)	*p* > 0.05	75 (24.3)	234 (75.7)	>0.05
Temporary job	29 (22.5)	100 (77.5)		14 (20.0)	56 (80.0)		15 (25.4)	44 (74.6)	
Disabled or retired	12 (24.0)	38 (76.0)		3 (16.7)	15 (83.3)		9 (28.1)	23 (71.9)	
Unemployed	210 (21.4)	773 (78.6)		51 (13.8)	319 (86.2)		159 (25.9)	454 (74.1)	
Subjective assessment of monthly income									
Sufficient to cover all living needs plus may save a certain amount	5 (27.8)	13 (72.2)	>0.05	1 (25.0)	3 (75.0)	*p* > 0.05	4 (28.6)	10 (71.4)	>0.05
Sufficient to cover all living needs	41 (22.9)	138 (77.1)		6 (16.2)	31 (83.8)		35 (83.8)	107 (75.4)	
Sufficient to cover basic needs only	179 (21.4)	658 (78.6)		51 (16.8)	252 (83.2)		128 (24.0)	406 (76.0)	
Not sufficient to cover even the basic needs	84 (20.3)	331 (79.8)		31 (14.9)	177 (85.1)		53 (25.6)	154 (74.4)	
Difficult to say	49 (29.2)	119 (70.8)		11 (21.2)	41 (78.8)		38 (32.8)	78 (67.2)	
Subjective health state									
Fair	143 (25.2)	424 (74.8)	>0.05	43 (21.5)	157 (78.5)	>0.05	100 (27.3)	267 (72.7)	>0.05
Rather fair	102 (20.2)	402 (79.8)		26 (14.4)	155 (85.6)		76 (23.5)	247 (76.5)	
Neither fair nor poor	74 (20.0)	297 (80.0)		22 (15.2)	123 (84.8)		52 (23.0)	174 (77.0)	
Rather poor	29 (22.3)	101 (77.7)		6 (10.7)	50 (89.3)		23 (31.1)	51 (68.9)	
Poor	10 (22.2)	35 (77.8)		3 (13.6)	19 (86.4)		7 (30.4)	16 (69.6)	
Number of health problems									
None	61 (28.0)	157 (72.0)	>0.05	23 (27.1)	62 (72.9)	>0.05	38 (28.6)	95 (71.4)	>0.05
1–3 health problems	192 (22.0)	679 (78.0)		46 (14.3)	276 (85.7)		146 (26.6)	403 (73.4)	
4–6 health problems	87 (20.1)	346 (79.9)		26 (16.0)	136 (874.0)		61 (22.5)	210 (77.5)	
≥7 health problems	18 (19.0)	77 (81.0)		5 (14.3)	30 (85.7)		13 (21.7)	47 (78.3)	
Awareness of ETS-associated health risks									
Yes	242 (23.3)	795 (86.7)	<0.05	63 (16.9)	310 (83.1)	>0.05	179 (27.0)	485 (73.0)	>0.05
No	116 (20.0)	464 (80.0)		37 (16.0)	194 (84.0)		79 (22.6)	270 (76.3)	
ETS exposure									
At home (yes; at home only and yes; both at work and at home)	31 (9.9)	282 (90.1)	0.0001	12 (7.7)	144 (92.3)	0.001	19 (12.1)	138 (87.9)	<0.001
Yes, at work only	18 (24.0)	57 (76.0)		10 (29.4)	24 (70.6)		8 (19.5)	33 (80.5)	
Yes, in other places only	80 (23.5)	261 (76.5)		26 (18.7)	113 (81.3)		54 (26.7)	148 (73.3)	
None	229 (25.8)	659 (74.2)		52 (18.9)	223 (81.1)		177 (28.9)	436 (71.1)	
ETS exposure (total number of hours per day)									
None	72 (20.4)	281 (79.6)	0.0000	28 (17.4)	133 (82.6)	<0.001	44 (22.9)	148 (77.1)	>0.05
To 1 h	72 (20.4)	281 (79.6)		28 (17.4)	133 (82.6)		44 (22.9)	148 (77.1)	
1–5 h	16 (10.7)	133 (89.3)		5 (6.2)	76 (93.8)		11 (16.2)	57 (83.8)	
5–8 h	9 (12.9)	61 (87.1)		3 (7.5)	37 (92.5)		6 (20.0)	24 (80.0)	
More than 8 h	12 (9.9)	109 (90.1)		9 (9.5)	86 (90.5)		3 (11.5)	23 (88.5)	

* *p*-value, Mantel-Haenszel chi-squared test.

**Table 2:** Odds ratios (OR) and 95% confidence intervals (CI) for lack of implementation of complete smoke-free home rules and selected socio-demographic characteristics in men (n = 526) and women (n = 1091) of socially-disadvantaged residents of Piotrkowski District.

Characteristic	Total n = 1617				
		Univariable Logistic Regression		Multivariable Logistic Regression ^a^	
	n %	OR	95% CI	OR	95% CI
Gender					
Male	419 (79.7)	1.17	0.91–1.51		
Female	840 (77.0)	1.00	reference		
Smoking status					
Smoker	504 (83.4)	1.72 ***	1.33–2.23	1.71 ***	1.32–2.21
Non-smoker	755 (74.5)	1.00	reference	1.00	reference
Age (years)					
<30	133 (73.5)	1.00	reference		
30–39	550 (79.4)	1.39	0.95–2.03		
40–49	422 (77.9)	1.27	0.86–1.87		
50–59	154 (76.6)	1.18	0.74–1.89		
Subjective assessment of living conditions					
Fair	124 (71.7)	1.00	Reference		
Rather fair	452 (77.1)	1.33	0.91–1.96		
Neither fair nor poor	567 (78.6)	1.45 *	1.00–2.12		
Rather poor, poor, difficult to say	116 (84.7)	2.18 **	1.23–3.86		
Education					
Primary	362 (80.8)	1.82 *	1.08–3.08		
Vocational	421 (78.0)	1.52	0.91–2.54		
Secondary	417 (76.5)	1.40	0.84–2.34		
High	58 (69.9)	1.00	Reference		
Employment status					
Permanent job	348 (76.5)	1.00	Reference		
Temporary job	100 (77.5)	1.06	0.66–1.69		
Disabled or retired	38 (76.0)	0.97	0.49–1.93		
Unemployed	773 (78.6)	1.13	0.87–1.48		
Awareness on environmental tobacco smoke health consequences					
Yes	795 (76.7)	1.00	Reference	1.00	Reference
No	464 (80.0)	1.30 *	1.01–1.67	1.28 *	1.00–1.65
Subjective assessment of monthly income					
Sufficient to cover all living needs plus may save a certain amount	13 (72.2)	1.00	Reference		
Sufficient to cover all living needs	138 (77.1)	1.29	0.44–3.85		
Sufficient to cover basic needs only	658 (78.6)	1.41	0.50–4.02		
Not sufficient to cover even the basic needs	331 (79.8)	1.52	0.53–4.37		
Difficult to say	119 (70.8)	0.93	0.32–2.76		
Subjective health state					
Fair	424 (74.8)	0.85	0.41–1.76		
Rather fair	402 (79.8)	1.13	0.54–2.36		
Neither fair nor poor	297 (80.0)	1.15	0.54–2.43		
Rather poor	101 (77.7)	1.00	0.44–2.43		
Poor	35 (77.8)	1.00	Reference		
Cohabitation with partner and/or family					
No (living alone)	201 (78.5)	1.00	Reference		
Yes	1058 (77.7)	0.96	0.69–1.32		

^a^ Fully-adjusted model including all statistically significant characteristics; * *p* ≤ 0.05; ** *p* ≤ 0.01; *** *p* ≤ 0.001.

## Supplementary Materials

The following are available online at http://www.mdpi.com/1660-4601/14/4/447/s1, Table S1: SM1.
